# Obstetric care in rural critical access hospitals: A domestic application of the World Health Organization Emergency Obstetric Care framework in rural communities

**DOI:** 10.1111/jrh.70037

**Published:** 2025-05-24

**Authors:** Annie L. Glover, Diane Brown, Carly Holman, Megan Nelson

**Affiliations:** ^1^ Rural Institute for Inclusive Communities University of Montana Missoula Montana USA; ^2^ School of Public Health University of Alabama at Birmingham Birmingham Alabama USA; ^3^ School of Public & Community Health Sciences University of Montana Missoula Montana USA

**Keywords:** emergency care, maternity care, obstetrics, rural hospitals

## Abstract

**Purpose:**

Pregnancy‐related mortality has increased steadily over the last 30 years in the United States; during the same period, rural communities have lost access to care as rural hospitals and obstetric units have shut their doors. Rural critical access hospitals (CAHs) are often the only option for a pregnant person in a rural community needing emergency care. This study aimed to apply a uniform assessment of the capacity of hospitals that do not have obstetric units to meet the emergency obstetric care needs of the rural communities they serve, with the goal of facilitating ongoing obstetric emergency readiness assessments that can be used in the rural context.

**Methods:**

The study team conducted facility assessments across Montana's statewide system of hospital care. The Centers for Disease Control and Prevention (CDC) Levels of Care Assessment Tool (LOCATe) was used in hospitals with an obstetrics unit (*N* = 25). The team adapted the World Health Organization (WHO) Emergency Obstetric Care (EmOC) framework to assess readiness in hospitals without an obstetrics unit (*N* = 34) but with Emergency Medical Treatment and Labor Act (EMTALA)‐based obligations to patients presenting to emergency departments with obstetric emergencies.

**Findings:**

None of the responding hospitals without obstetric units met criteria indicating readiness to provide comprehensive emergency obstetric care (CEmOC), and only one hospital met criteria indicating readiness to provide basic emergency obstetric care (BEmOC).

**Conclusion:**

Significant work must be done to bring CAHs up to a level of readiness where they can safely and effectively screen, stabilize, and transfer or accept an obstetric emergency. The WHO EmOC framework can provide a starting point for assessing the capacity of hospitals without obstetric units, but a standardized assessment, such as LOCATe, should be developed to improve readiness for obstetric emergencies.

## INTRODUCTION

Maternal mortality in the United States has been on an upward trajectory since the year 2000^1^; already higher than any other Organisation for Economic Co‐Operation and Development (OECD) country, the maternal mortality ratio (MMR) in the United States continues to rise, while peers around the world have experienced declines in maternal deaths in recent years.^1^, ^2^ The magnitude of the disparity is primarily driven by significantly higher risks experienced by sociodemographic groups that have historically experienced inequities in health care access and care quality. Individuals living in rural communities represent one important and understudied group that has carried an outsized burden of poor maternal health outcomes.^3^, ^4^ In a study of pregnancy‐related deaths, Merkt et al. found that rurality was associated with an increased risk for death for the general population as well as across all racial and ethnic subgroups.^5^ Well‐documented racial disparities for Indigenous and Black populations are further compounded by resource constraints experienced by residents of rural communities.^4^, ^5^, ^6^ These data have been solidified by more recent analyses of pregnancy‐related deaths from the Pregnancy Mortality Surveillance System and U.S. Maternal Mortality Review Committees.^7^, ^8^, ^9^, ^10^ These findings indicate that maternal health disparities should be studied through an intersectional lens.

Health disparities experienced by rural communities are driven by geographic challenges, such as isolation and distance to urban centers, and social determinants of health, such as poverty and access to specialists and higher levels of care. Individuals who must travel great distances to receive risk‐appropriate care will experience worse health outcomes due to care delays and disruptions in care continuity.^11^, ^12^, ^13^, ^14^ The number of counties without any obstetric providers, such as certified nurse midwives and obstetrician/gynecologists (OBGYNs), or services such as birth centers and hospitals with obstetric units increased by 5% from 2020 to 2022.^15^ Between 2005 and 2023, 184 rural hospitals closed, and between 2011 and 2021, 267 obstetrics units in rural hospitals closed nationwide.^16^, ^17^ These rural closures, which have been attributed primarily to financial pressures and inadequate insurance reimbursements, increase the risk of preterm births, births outside the hospital, or births at a hospital without obstetric services.^18^ American Indian/Alaska Native (AI/AN) people are 20 times more likely than White people to deliver at a hospital without obstetric services.^19^ In Montana, AI/AN people who live in rural areas or on remote Federal Indian Reservations also have less access to complex or specialty obstetric care, have to travel further to receive care, and are more likely to seek care at a nonlocal obstetric unit to give birth, underscoring the intersection of race and geography in these extreme health disparities.^19^, ^20^ Long drive times in rural states like Montana can be exacerbated by poor weather conditions, remote areas, and infrastructure challenges, making accessing a hospital with risk‐appropriate care more daunting.

For rural residents who live far from birthing centers, emergency departments (EDs) in critical access hospitals (CAHs) are often a last resort option for emergency obstetric care, which includes care provided in an ED setting for pregnant and postpartum patients presenting with acute complications of pregnancy or active labor. The Emergency Medical Treatment and Labor Act (EMTALA) requires that EDs stabilize and treat anyone with an emergency medical condition regardless of their ability to pay or insurance status.^21^ EMTALA requires hospitals to provide a screening examination that is medically appropriate for the patient. For pregnant patients, this examination determines if the person is in active labor or has an emergency medical condition.^22^ If the hospital does not have the capacity to stabilize the patient, EMTALA allows the transfer of the patient; however, if the patient is in active labor, the patient may only be transferred if the hospital determines that the benefits outweigh the risks to both the pregnant patient and fetus. Despite this federal law, the United States does not have a set of consensus guidelines that provide a standard for assessing hospital readiness to provide obstetric care in EDs.^23^ This study contributes to the body of literature on emergency obstetric preparedness, and it also serves as a call to action for the development of guidelines and tools that rural hospitals can deploy in their efforts to build capacity for high‐quality emergency care that meets the needs of pregnant and in‐labor patients presenting to their EDs.

### Framework

The World Health Organization (WHO) has set forth guidelines that are broadly applied in low‐ and middle‐income countries (LMICs) on the provision of emergency obstetric care in nonbirthing facilities, based on the clinical capacity of health care facilities to provide either basic emergency obstetric care (BEmOC) or comprehensive emergency obstetric care (CEmOC). The WHO Emergency Obstetric Care (EmOC) package assesses facilities’ capacity for the purposes of prioritizing system improvements to treat emergency obstetric patients for leading causes of maternal morbidity and mortality, including hemorrhage, prolonged or obstructed labor, postpartum sepsis, preeclampsia or eclampsia, ectopic pregnancy, ruptured uterus, intrapartum newborn distress, and complication of abortion or miscarriage.^24^, ^25^

### WHO indicators

The WHO's *Monitoring Emergency Obstetric Care: A Handbook* contains a list of nine life‐saving clinical services, or signal functions (identified as indicators in this study), concerning hospital readiness to respond to obstetric emergencies.^24^ It details the necessary indicators to provide BEmOC and CEmOC (Table 1).^24^ CEmOC consists of the seven indicators of BEmOC, plus two additional components: the capacity to perform surgery (e.g., cesarean section) and a blood transfusion. For both definitions of BEmOC and CEmOC, all indicators must be available at hospitals 24 h a day, 7 days a week, and the quality of care remains the same regardless of the day or hour.^24^

**TABLE 1:** World Health Organization Emergency Obstetric Care indicators.

Basic Emergency Obstetric Care services (BEmOC)	Comprehensive Emergency Obstetric Care services (CEmOC)
1. Administer parenteral antibiotics	Perform indicators 1–7, plus:
2. Administer uterotonic drugs	8. Perform surgery (e.g., cesarean section)
3. Administer parenteral anticonvulsants for pre‐eclampsia and eclampsia	9. Perform blood transfusion
4. Manually remove the placenta	
5. Remove retained products	
6. Perform assisted vaginal delivery	
7. Perform basic neonatal resuscitation	

## METHODS

As part of a larger needs assessment that was funded by the Health Resources and Services Administration Maternal Health Innovation grant awarded to Montana in 2019, the study team conducted an assessment of facility readiness to provide obstetric care based on levels of risk, using the Centers for Disease Control and Prevention Levels of Care Assessment Tool (LOCATe).^26^, ^27^ LOCATe provides a standardized assessment that classifies facilities with a labor and delivery department into levels based on staffing, volume of services, and equipment. It supports regional and system development of risk‐appropriate care.^27^, ^28^, ^29^, ^30^, ^31^, ^32^ The levels of maternal and neonatal care, as defined in Table 2 and assessed in LOCATe, align with the 2012 guidelines and policy statements from the American Academy of Pediatrics and the 2019 and 2023 guidelines from the American College of Obstetricians and Gynecologists (ACOG) and Society for Maternal and Fetal Medicine (SMFM).^29^, ^31^, ^32^

**TABLE 2:** Maternal and neonatal levels of care.^26^, ^29^, ^31^, ^32^

Hospital‐level	Maternal	Neonatal
<Level I^a^	Does not meet ACOG/SMFM minimum level designation	–
Level I	Basic care	Well newborn nursery
Level II	Specialty care	Special nursery care
Level III	Subspecialty care	Newborn Intensive Care Unit (NICU)
Level IV	Regional perinatal health center	Regional NICU

^a^ The LOCATe assessment considers facilities <Level I if they do not meet the ACOG/SMFM floor requirements for Level I. The <Level I facilities in Montana operate at Level I.

Initially, the team intended to use the LOCATe instrument to assess all Montana hospitals. However, during stakeholder review of the instrument, it became immediately apparent that the LOCATe assessment was not applicable in rural CAHs or other hospitals that do not have a dedicated obstetrics department but that, nonetheless, handle deliveries and obstetric emergencies through their EDs. Given the essentially different nature of obstetric care provided in hospitals that do not have obstetrics units, the team decided to assess these facilities for emergency preparedness in obstetric care.

### Survey instrument

The research team adapted a survey developed by Kozhimannil et al., who used an adapted WHO EmOC framework for a national study within the United States to examine the capacity of rural hospitals without obstetrics services.^23^ The survey includes components of the WHO EmOC indicators and other measures of emergency obstetric capacity in rural hospitals without obstetric units. Kozhimannil et al. provided the response options “no, we lack necessary staff,” “no, we lack necessary equipment,” “no, we lack both staff and equipment,” and “don't know” for the nine EmOC indicators.^23^ They identified additional measures of emergency obstetric capacity from a literature review on emergency obstetric care in rural communities, and clinicians practicing in rural hospitals without obstetric units reviewed the survey.^23^ We adapted this survey to capture the unique challenges of emergency obstetric services in Montana and had three Montana‐based providers (one OBGYN physician, one Family Medicine physician, and one nurse) review the survey. Based on their clinical guidance, we adapted the wording, as seen in the Supporting Information Appendix, and added two questions: “my hospital has the capacity to provide and interpret fetal heart tracing in an emergency setting,” and “my hospital has a plan or policy for an emergency cesarean in the case of a life‐threatening obstetrical hemorrhage or profound fetal distress.” The survey included closed‐ended and open‐ended questions about hospital characteristics, staffing, training, transport, medical products, equipment, technology, EmOC indicators, perinatal services, and emergent event history.

### Data and study population

We sent an email invitation to the Director of Nursing at all hospitals in Montana without obstetrics services (*N* = 34) to participate in the Emergency Obstetrics Services Survey. Data collection occurred using REDCap^33^, ^34^ from October 18, 2021, to December 10, 2021. The survey remained open for 8 weeks, and hospitals received email reminders weekly. Midway through the recruitment period, we called hospitals that had not completed the survey. As part of the broader ongoing needs assessment to which this study was attached, we conducted a data validation process with hospitals from April 10, 2023, to July 17, 2023, as part of an extension of the assessment that helped to refine program goals and strategies further. Facilities reviewed their 2021 responses to the WHO EmOC indicators, confirmed data accuracy, or made updates based on their current capacity. We also added two WHO EmOC indicators missing from the 2021 survey, “administer intravenous or injection antibiotics to mothers and infants” and “perform surgery (e.g., cesarean section).” In addition to conducting the data validation process with the prior participants, we contacted the facilities that did not respond to the survey in 2021 and invited them to participate. The University of Montana Institutional Review Board (IRB) determined that this study did not need IRB approval as it did not involve human subjects research.

### Data analysis

Utilizing the results from the Montana LOCATe assessment,^26^ the research team calculated driving distances in miles using Google Maps^35^ from the nonbirthing hospitals to the nearest birthing facility for each maternal and neonatal care level using facility‐reported addresses. The research team used both the 2013 urban–rural classification scheme for counties (Table 3) as defined by the National Center for Health Statistics (NCHS) and the 2023 Rural–Urban Continuum Codes (RUCC) as defined by the Economic Research Service of the U.S. Department of Agriculture (Table 4).^36^, ^37^, ^38^

**TABLE 3:** The 2013 National Center Health Statistics urban–rural classification scheme.^37^

Urban/rural designation	Classification	Description
Metropolitan (urban)	Large central metro	Counties in metropolitan statistical areas of 1 million population or more
	Large fringe metro	Counties in metropolitan statistical areas of 1 million or more population that do not qualify as large central
	Medium metro	Counties in metropolitan statistical areas of 250,000–999,999 population
	Small metro	Counties in metropolitan statistical areas of less than 250,000 population
Nonmetropolitan (rural)	Micropolitan	Micropolitan counties in a micropolitan statistical area
	Noncore	Noncore counties not in micropolitan statistical areas

**TABLE 4:** The 2020 Rural‐Urban Continuum Codes of the Economic Research Service.^38^

Urban/rural designation	Codes	Description
Metropolitan counties (urban)	1	Counties in metro areas of 1 million population or more
	2	Counties in metro areas of 250,000 to 1 million population
	3	Counties in metro areas of fewer than 250,000 population
Nonmetropolitan counties (rural)	4	Urban population of 20,000 or more, adjacent to a metro area
	5	Urban population of 20,000 or more, not adjacent to a metro area
	6	Urban population of 5000–20,000, adjacent to a metro area
	7	Urban population of 5000–20,000, not adjacent to a metro area
	8	Urban population of fewer than 5000, adjacent to a metro area
	9	Urban population of fewer than 5000, not adjacent to a metro area

For the 2013 urban–rural classification scheme, our study only used the three smallest designations: small metro, micropolitan, and noncore. The 2023 RUCC allow insight into each county's urbanization and adjacency to a metro area. Our study only required the smallest metro designation and two of the six nonmetro levels. We used both definitions to capture the nuance of rurality and to reflect some of the changes in Montana over the last decade.

The research team used Stata 18.0^39^ to conduct all quantitative analyses. We used descriptive analyses for hospital characteristics, interactions, and WHO EmOC capacity. Based on a previous study assessing WHO EmOC capacity,^40^ we tested our hypothesis that facilities would have higher EmOC indicator scores if they experienced more emergency obstetric events. Due to small sample sizes, we conducted this analysis and additional bivariate significance testing using two‐tailed Fisher's Exact tests to assess the association between various hospital characteristics, such as driving distance, rurality, hospital size, reservation adjacency, and a hospital's capacity for EmOC based on the presence of indicators. We selected these characteristics due to established literature indicating that they could influence a hospital's capacity for obstetric services.^4^, ^19^, ^20^, ^23^, ^40^, ^41^

We collapsed variables for Fisher's exact tests and some descriptive analyses to facilitate significance testing. This included variables on emergency obstetric events (originally reported as different ranges of events: 1–2, 3–4, 5–10, more than 10, or none; changed to had an event/did not have an event), EmOC indicators (originally reported as yes, no—lack staff, no—lack equipment, no—lack both staff and equipment, or don't know; changed to yes/no), and both rurality definitions. We collapsed RUCC and NCHS categories into metro and nonmetro.

All hospitals without obstetric services who completed the survey in the second round (*N* = 34) answered all the questions about the WHO EmOC indicators. A few facilities responded “don't know” to specific indicators, but the research team considered this a valid response within the survey. Among other questions the research team used in analyses (i.e., hospital characteristics, interactions, and resources), 4.5% had missing responses, not including those questions that not every hospital was asked due to skip logic. To determine missingness for analyses, we identified any survey response where at least one of the questions did not have a valid response.

## RESULTS

### Hospital characteristics

Of the 34 originally queried facilities without obstetrics units, 32 (94%) participated in the initial survey. In the follow‐up survey in 2023, we approached 35 facilities without obstetrics units, and 34 (97%) participated. All hospitals that completed the survey in 2021 are CAHs, and two are also designated Indian Health Service Units. In 2023, an urban hospital without an obstetrics unit and a CAH that had not completed the survey in 2021 also completed the survey in addition to the original group of CAHs without obstetric units. One CAH did not complete the study in 2021 or 2023.

The hospitals that completed the survey represented 31 of Montana's 56 counties (Table 5). Almost all hospitals reside in rural noncore counties (91.2%), according to NCHS 2013 definitions,^37^ and nearly half were nonadjacent, nonmetro (47.1%) based on 2023 RUCC.^38^ The number of acute care inpatient beds ranges from 2 to 25, with a median of 12.5. About half (44.1%) of surveyed nonbirthing hospitals previously had an obstetric unit, with a little less than half (40.0%) of them having closed within the last 20 years.

**TABLE 5:** Characteristics of participating hospitals.

2023 hospital characteristics (*N* = 34)	*n* (%)
Critical access hospitals^a^	33 (97.1)
Indian health service units^a^	2 (5.9)
Urban specialty hospital	1 (2.9)
Location	
County population (median [IQR])	5434.5 [2314–8878]
<2000 people	8 (23.5)
2001–5000 people	9 (26.5)
5001–8000 people	5 (14.7)
8001–11,000 people	7 (20.6)
> 11,000 people	5 (14.7)
Urban–rural NCHS 2013	
Noncore	31(91.2)
Micropolitan	1 (2.9)
Small metro	2 (5.9)
Urban–rural RUCC 2023	
Nonadjacent nonmetro, population <5000	16 (47.1)
Adjacent nonmetro, population <5000	12 (35.3)
Metropolitan designation, population <250,000	6 (17.7)
Hospital size	
Total number of beds (median [IQR])	12.5 [6–24]
2–10 acute care inpatient beds	17 (50.0)
11–20 acute care inpatient beds	7 (20.5)
21–25 acute care inpatient beds	10 (29.4)
Hospital previously had an obstetrics unit	15 (44.1)
Year of obstetric services loss	
Never had obstetric services	19 (55.9)
Within the last 5 years	2 (5.9)
6–10 years ago	1 (2.9)
11–20 years ago	3 (8.8)
21–30 years ago	3 (8.8)
31–40 years ago	3 (8.8)
More than 40 years ago	3 (8.8)

*Note*: Data are *n* (%) unless otherwise stated as median [IQR]. Not all categories add to 100% due to rounding.

Abbreviation: IQR, interquartile range.

^a^ A hospital can be both an Indian Health Service Unit and a Critical Access Hospital.

Most (85.3%) hospitals reported that they had the ability to administer magnesium sulfate for severe preeclampsia and eclampsia (Table 6), which ACOG and the SMFM recommend as both prophylaxis and treatment for pregnant patients with eclampsia and severe preeclampsia to prevent or reduce seizures^42^; to administer parenteral (intramuscular or intravenous) antibiotics (85.3%), which can significantly reduce the risk of maternal sepsis and death from sepsis^43^; and to perform basic neonatal resuscitation (82.4%), which the care team should initiate within 1 min of birth based on the results of the initial assessment of heart rate and respiration, potentially reducing poor short‐ and long‐term outcomes.^44^

**TABLE 6:** Hospital capacity in responding to obstetric emergencies using WHO Emergency Obstetric Care indicators.

Hospital has the capacity to… (*N* = 34)	*n* (%)	WHO indicator type
Administer magnesium sulfate for severe preeclampsia and eclampsia	29 (85.3)	BEmOC
Administer injection or intravenous antibiotics to both mother and infant	29 (85.3)	BEmOC
Perform basic neonatal resuscitation	28 (82.4)	BEmOC
Conduct blood transfusion	26 (76.5)	CEmOC
Administer uterotonic drugs	18 (52.9)	BEmOC
Perform assisted vaginal delivery with a soft cup vacuum extractor	5 (14.7)	BEmOC
Manually remove a placenta	5 (14.7)	BEmOC
Remove retained products of delivery	4 (11.8)	BEmOC
Perform surgery	0 (0.0)	CEmOC
Additional non‐WHO indicator*s*		
Hospital had at least basic ultrasound capabilities for obstetric emergencies^a^	12 (37.5)	N/A
Hospital could provide and interpret fetal heart tracing	12 (35.3)	N/A
Montana's responding hospital capacity		
Capacity to perform all assessed CEmOC indicators	0 (0.0)	N/A
Capacity to perform all assessed BEmOC indicators	1 (2.9)	N/A
Number of indicators performed (mean)	4.2	N/A

*Note*: Data are *n* (%) unless otherwise stated as mean. Not all categories add to 100% due to rounding.

^a^ *N* = 32, two responses missing.

Fewer hospitals had the capacity to administer uterotonic drugs (55.2%) like oxytocin, which are administered parenterally during labor or immediately after to prevent and treat obstetric hemorrhage by aiding in the contraction of the uterus.^24^, ^45^ Most responding hospitals reported not having the capacity to perform several components of WHO BEmOC that may require specialized equipment and/or training. Only five facilities (14.7%) reported having the capacity to perform assisted vaginal delivery with a soft cup vacuum extractor or manually remove a placenta, and only four (11.8%) had the capacity to remove retained products of conception (RPOC) (this can be done using dilation and curettage or manual vacuum extraction, among other methods).^24^ “RPOC” is a medical term that refers to pregnancy tissue that can remain from delivery (either vaginal or cesarean), miscarriage, or induced abortion, which can lead to infection or postpartum hemorrhage if not removed.^46^, ^47^

For the additional CEmOC criterion, no hospital had the capacity to perform surgery like an emergency cesarean section (0.0%). In contrast, most facilities had the capacity to conduct a blood transfusion (76.5%), which may be indicated in cases of obstetric hemorrhage or gynecological emergencies such as ectopic pregnancy, ruptured uterus, or miscarriage.^24^ Hospitals frequently selected the “lack both the staff and equipment” response as the reason why they did not have the capacity to perform one of the indicators (see Supporting Information Appendix). This response could mean that the hospital lacks the necessary staff, that the current staff lacks appropriate training or experience, or that the hospital is missing equipment, lab services, and/or blood products.

Although the WHO indicators do not specify blood product availability, our survey asked about the availability of packed red blood cells (PRBCs), fresh frozen plasma (FFP), platelets, and cryoprecipitate (used if clinical coagulopathy is present) based on ACOG's Massive Transfusion Protocol (MTP) recommendations.^48^ Nearly all (80.6%) responding hospitals had some quantity of PRBCs available, but quantities varied (Table 7). Other types of blood products were less common, with 11 (35.5%) reporting that they had FFP and only one hospital (3.3%) using platelets and cryoprecipitate. When hospitals did not have blood transfusion capacity, they reported using several mechanisms to get blood products in emergencies, including courier, hospital staff picking it up at either the closest hospital or blood bank, or, most frequently, delivery from emergency transport services (either by ambulance or flight team). Overall, none (0.0%) of the hospitals reported “full readiness” for obstetric emergencies, which encompasses CEmOC (Table 6), and only one hospital (2.9%) had readiness for BEmOC.

**TABLE 7:** Blood product availability reported by Montana hospitals without obstetric units.

Available blood products (*N* = 31)	*n* (%)
Units of packed red blood cells (PRBCs)	
0 units	6 (19.4)
1–2 units	10 (32.3)
3–4 units	5 (16.7)
5–6 units	4 (16.1)
7 or more units	6 (19.4)
Units of fresh frozen plasma (FFP)	
0 units	20 (64.5)
1 unit	1 (3.2)
2 units	7 (22.6)
3 or more units	3 (9.7)
Units of platelets^a^	
0 units	29 (96.7)
1 unit	1 (3.3)
Units of cryoprecipitate^a^	
0 units	29 (96.7)
1 unit	1 (3.3)

*Note*: Three hospitals skipped all questions on blood products.

^a^ *N* = 30, one additional response missing.

As previously described, we supplemented the WHO indicators with additional questions on capacity. More than a third (35.3%) had the capacity to provide and interpret fetal heart tracing. Only one hospital (2.9%) had a plan or policy for an emergency cesarean in the case of a life‐threatening obstetric hemorrhage or profound fetal distress (Table 9). In addition, almost half of responding hospitals (46.9%) reported not having ultrasound capabilities for obstetric emergencies.

We explored these challenges for hospital capacity by hospital attributes to determine if a hospital's attributes, including hospital size, driving distance to a hospital with an obstetric unit, reservation adjacency, and rurality, affected their capacity for EmOC. Based on these attributes, we found no significant difference in a hospital's capacity for EmOC (Table 8).

**TABLE 8:** WHO indicator capacity by hospital attributes.

	WHO indicator capacity		
	*n* (%)		
Hospital attributes (*N* = 34)	Six or less indicators	Seven or more indicators	Two‐sided Fisher's exact
Hospital size			0.485
10 or fewer beds	17 (53.1)	0 (0.0)	
11 or more beds	15 (46.9)	2 (100.0)	
Rurality (NCHS 2013)			1.000
Metro (small metro)	2 (6.3)	0 (0.0)	
Nonmetro (micropolitan or noncore)	30 (93.8)	2 (100.0)	
Rurality (RUCC 2023)			1.000
Metro	6 (18.8)	0 (0.0)	
Nonmetro	26 (81.3)	2 (100.0)	
Reservation adjacency			0.485
Adjacent to a reservation	15 (46.9)	2 (100.0)	
Not adjacent to a reservation	17 (53.1)	0 (0.0)	
Driving distance to closest hospital with obstetrics unit			0.214
<50 miles	14 (43.8)	2 (100.0)	
≥50 miles	18 (56.3)	0 (0.0)	

*Note*: Data are n (%). Not all categories add to 100% due to rounding.

To recognize the need for EmOC readiness, the survey included questions about obstetric or gynecological events in the 2 years prior to taking the survey. Most hospitals had fewer than 10 obstetric or gynecological events. Of the different types of these events, 79.4% of the facilities reported gynecological emergencies, with one hospital reporting 25 (Table 9). Other gynecological emergencies can encompass but are not limited to hemorrhaging from ovarian cysts, abscesses, or other causes; ectopic pregnancies; miscarriages; pelvic inflammatory disease; or trauma.^49^ Some of these emergencies may require the facility to remove RPOC or perform surgery.

**TABLE 9:** Emergency obstetric events over a 2‐year period and hospital response as reported by Montana hospitals without an obstetric unit.

Emergency obstetric events & response (*N* = 34)	*n* (%)
Obstetric or gynecologic events	
Facility had a gynecological emergency	27 (79.4)
Facility had a pregnant person present in labor	26 (76.5)
Facility had a postpartum emergency	19 (55.9)
Facility had an emergency room birth^a^	16 (48.5)
Facility expressed concern about providing EmOC due to their frequency and facility's lack of experience	16 (47.1)
Facility experienced a close call or other unanticipated birth outcome	12 (35.3)
Policies, procedures, and processes for emergency obstetric events	
Facility had a plan or policy for an emergency cesarean in the case of a life‐threatening obstetric hemorrhage or profound fetal distress	1 (2.9)
Facility has a massive transfusion protocol^b^	12 (38.7)
Facility has a transport protocol or policy for pregnancy patients^c^	9 (30.0)
Facility has a formal agreement with another hospital that has OB services^b^	12 (38.7)
Facility reported holding debriefs following emergency obstetric events^d^	16 (50.0)

Abbreviation: EmOC, Emergency Obstetric Care

^a^ *N* = 33, one response missing.

^b^ *N* = 31, three responses missing.

^c^ *N* = 30, four responses missing.

^d^ *N* = 32, two responses missing.

People presenting to the hospital in labor was the most frequent obstetric occurrence (76.5%), with two hospitals having 50 cases each. Almost half (48.5%) of the hospitals without obstetric units reported emergency room births, with one hospital reporting 16. Both gynecological emergencies and those in labor may require blood transfusions. Although 76.5% of hospitals reported having the ability to perform blood transfusions, 38.5% had only 1–2 units of PRBCs available. ACOG recommends a minimum of 4 units of O‐negative PRBCs, with the ability to obtain 6 units in case of obstetric hemorrhage.^48^ ACOG also recommends other blood products (FFP, platelets, and cryoprecipitate) as part of their MTP, in addition to recommending that facilities have their own MTP. Slightly more than a third (38.7%) of responding hospitals (with three missing responses) reported having one.

Over a third (35.3%) of facilities reported experiencing a close call or other unanticipated adverse birth outcome during one of these events. Nearly half (47.1%) of the hospitals described concern about their hospital's capacity to provide emergency obstetric services, specifically because of the infrequency of emergency obstetric events and their hospital's lack of experience in response to them. We completed additional analyses to determine if hospitals without obstetric units that had seven or more WHO EmOC indicators had experienced more of these interactions (i.e., a pregnant person presenting in labor, emergency room birth, or postpartum emergency), and none of these associations were statistically significant with the small numbers present (Table 10).

**TABLE 10:** WHO indicator capacity by hospital interactions.

	WHO indicator capacity		
	*n* (%)		
Hospital interactions (*N* = 34)	Six or less indicators	Seven or more indicators	Two‐sided Fisher's exact
Pregnant person presented in labor			1.000
Yes	24 (75.0)	2 (100.0)	
No	8 (25.0)	0 (0.0	
Emergency room birth			0.227
Yes	14 (45.2)	2 (100.0)	
No	17 (54.8)	0 (0.0)	
Postpartum emergency			0.492
Yes	17 (53.1)	2 (100.0)	
No	15 (46.9)	0 (0.0)	
Gynecological emergency			1.000
Yes	25 (78.1)	2 (100.0)	
No	7 (21.9)	0 (0.0)	
Hospital experienced a close call or unanticipated birth outcome			0.118
Yes	10 (31.3)	2 (100.0)	
No	22 (68.8)	0 (0.0)	

In situations where hospitals do not have the capacity to provide emergency obstetric care, they are often required to transport patients emergently to the closest hospital offering risk‐appropriate care. As described in the methods, we calculated distances to care based on LOCATe levels of care. In over half (52.9%) of responding hospitals, a patient must travel or be transported more than 75 miles to reach a Maternal Level II or higher facility from the assessed hospital without an obstetrics unit (Table 11). If the fetus or infant will require intensive care (neonatal intensive care unit), 70.6% of hospitals without obstetric units would require arranging a transfer of 75 miles or more for the patient to reach a Neonatal Level III facility.

**TABLE 11:** Calculated driving distance to hospitals by maternal and neonatal levels of care.

Calculated driving distance care (*N* = 34) (in miles)	Median [IQR]
Maternal Level I	56.1 [41.0–71.9]
Maternal Level II	77.1 [57.4–97.2]
Maternal Level III or IV	218.5 [154.5–278.5]
Neonatal Level I	50.4 [38.9–61.8]
Neonatal Level II	58.9 [44.1–91.6]
Neonatal Level III	98.9 [62.7–193]

Abbreviation: IQR, interquartile range.

## DISCUSSION

The WHO EmOC indicators have been historically used in LMICs, but in this study, we show that they can indicate a hospital's readiness for obstetric emergencies within a rural CAH in the United States. Our findings show that most of this state's rural hospitals without obstetric units do not have readiness for obstetric emergencies, and providers feel concerned and unprepared when an obstetric emergency walks through their door.

Our study highlights that not only were hospitals without obstetrics units facing obstetric and gynecological emergencies, but they often did not have the appropriate materials available for those emergencies, especially blood supply for transfusions. The high incidence of unintentional injuries in Montana frequently exacerbates the issue of blood supply shortages.^50^ Often, the nonobstetric emergencies that occur in noncore counties result in fatalities and have higher rates of nonfatal hospitalization and visits to the emergency room.^50^ These nonobstetric emergencies may require blood transfusion and parenteral antibiotics, demanding the facility's limited resources. They could also take priority for transfers, potentially utilizing the only transport and staff available due to the high acuity of the accident. Within this study, respondents reported that the blood supplies in their EDs are often used for other emergencies, including traumas and gastrointestinal bleeds. These types of emergencies are the norm within the CAHs of Montana, whereas obstetric emergencies are more infrequent and lead to stress for the provider.

There are several hospital interventions to improve readiness for obstetric emergencies. To improve the outcomes of pregnant people, nonbirthing hospitals need to feel confident in handling basic obstetric emergencies. Hospitals frequently described in the assessed EmOC indicators a need for staff and equipment, as well as increased training arising from concerns about responding to obstetric emergencies. A study by Kozhimannil et al. found that staff at nonbirthing facilities reported a need for additional resources and training to handle obstetric emergencies.^23^ Periodic and repeated training can provide nonbirthing hospitals with the skills and capacity to treat and stabilize pregnant patients until they can arrange transport to risk‐appropriate care.^51^ An educational standard for many clinical subspecialties, in situ simulation training provides clinicians with opportunities to practice and hone skills through practical experiences in mobile units or their hospitals. Recent studies have found that hospitals prefer the hands‐on experience of simulation training.^52^, ^53^, ^54^ With mobile simulation units, rural hospitals with limited budgets have more opportunities to access simulation training that can increase or maintain obstetric skills.^52^, ^53^, ^54^ To add to this knowledge, the Alliance for Innovation on Maternal Health (AIM) supports hospitals in low‐resource settings or without obstetric units with the AIM Emergency Readiness Resource Kit, using resources to support readiness in an obstetric emergency.^55^

In addition to training, our findings found that surveyed hospitals had few policies, procedures, or processes in place to handle obstetric emergencies. A recent study in Montana found that few staff were aware of policies and procedures for transporting obstetric emergencies, compounding the issue.^56^ Having them in place is a step in improving outcomes in these emergent situations, and ACOG and AIM have recommended it.^55^, ^57^, ^58^ In addition, the National Advisory Committee on Rural Health and Human Services recommends the development of guidelines and implementation of protocols in an effort to respond to obstetric complications in hospitals with and without obstetric services.^59^

In a study that was conducted as a qualitative follow‐up to the assessment presented here, Fertaly et al. collected narratives from ED staff at CAHs that illustrate the acute and often intense circumstances in which these facilities are managing obstetric emergencies, with stabilization and transport processes often complicated by weather and underresourced, volunteer‐based support services. They further discovered that hospitals without obstetric units did not have a well‐coordinated plan with obstetrics hospitals, often due to the lack of a perinatal regionalized system of care.^56^ When CAHs do not have EmOC readiness, relationships need to be established with hospitals that can provide risk‐appropriate care. The development of regional perinatal networks would help address the need for higher obstetrical services, distance to care, and limited resources while maintaining quality services.^27^, ^60^, ^61^, ^62^, ^63^ Perinatal regionalization improves referral relationships, effective coordination, and the timely provision of risk‐appropriate care.

### Assessing readiness

Other studies assessing emergency obstetric capacity encountered similar difficulties with the WHO EmOC framework that Montana experienced when assessing maternal health systems.^25^, ^40^ Specifically, distance to care is an incomplete measure of access. It does not capture all potential barriers a pregnant person or facility may face when attempting to obtain risk‐appropriate care due to geographic, climate, and/or other accessibility challenges. Gausman et al. reflected that only assessing the indicators’ availability is insufficient to capture EmOC functionality.^40^ Their study suggested adding the Availability, Accessibility, Acceptability, and Quality framework in addition to the WHO EmOC framework, assessing not only availability but also accessibility, acceptability, and quality of the WHO EmOC functions. By combining the two frameworks, their study suggests that the assessment ensures that facilities not only meet either BEmOC or CEmOC functions but also provide these functions across geographic and sociodemographic divides; they are performed using culturally informed and respectful care and meet all quality standards.^40^

Creating a standardized tool like LOCATe to assess the capacity of hospitals without obstetric units is critical as obstetric units and hospitals increasingly close, and more pregnant people may seek care at one of these facilities. A standardized tool will allow hospitals to understand their capacity and need for training, process development, and relationship building through perinatal regionalization.

## LIMITATIONS

The distances to care were measured using Google Maps to inform the calculations between hospitals without obstetric units and birthing facilities for patient transfers. This may not represent the preferred routes of transport teams, the different modes of transport (i.e., ambulance, fixed‐wing, or helicopter), or the challenges often present during transport in Montana. In addition, our distance calculations are informative for patient transfer, but they do not give us any information on the distances patients travel from their homes to the hospital.

While the survey was comprehensive, it does not cover all potential services that may be needed during an obstetric emergency. A strength of this study was the response rate, with a 97.1% response from the hospitals without obstetric units in Montana; however, the research team designed the survey as a self‐assessment of facilities and not a formal audit of processes and data. This design makes the study vulnerable to information bias.

## CONCLUSION

As a response to the needs of rural EDs, this study makes an important contribution to the field's understanding of system‐level barriers to improving maternal health for all populations and communities. In the United States, there is no current assessment of readiness or capacity for obstetric emergencies in hospitals without obstetric units that has been standardized to risk‐appropriate perinatal care standards. The findings from this study underscore the importance of this issue in these hospitals and the impact that national engagement on emergency obstetric readiness can make toward addressing the U.S. maternal health crisis as that crisis has materialized in rural communities. Although the WHO EmOC indicators are typically used in LMICs, they provide a useful framework that can be adapted for the domestic rural setting to the context of the U.S. CAH system. A CAH without an obstetric unit may be the only hospital available for pregnant patients, without regard to weather, road conditions, or the distance from the patient's home. Policy‐level and facility‐level efforts to improve maternal health outcomes for rural populations are better equipped for success by actionable data gathered through replicable and comparable measurement guidelines, such as those proposed in this study, that help policymakers and funders prioritize strategic investments that will elevate care quality at each entry point and level of maternal health care.

## CONFLICT OF INTEREST STATEMENT

The authors declare no conflicts of interest.

## Supporting information



Supporting Information

## References

[1] Gunja MZ , Gumas ED , Williams RD II . The U.S. Maternal Mortality Crisis Continues to Worsen: An International Comparison . The Commonwealth Fund; 2022. doi:10.26099/8vem-fc65

[2] Tikkanen R , Gunja M , FitzGerald M , Zephyrin L . Maternal Mortality and Maternity Care in the United States Compared to 10 Other Developed Countries. The Commonwealth Fund; 2020.

[3] Rossen LM , Ahrens KA , Womack LS , Uddin SFG , Branum AM . Rural‐urban differences in maternal mortality trends in the US, 1999–2017: accounting for the impact of the pregnancy status checkbox. Am J Epidemiol. 2022;191(6):1030‐1039. doi:10.1093/aje/kwab300 35020799 PMC9177508

[4] Kozhimannil KB . Declining access to US maternity care is a systemic injustice. BMJ. 2023;382:p2038. doi:10.1136/bmj.p2038 37678911

[5] Merkt PT , Kramer MR , Goodman DA , et al. Urban‐rural differences in pregnancy‐related deaths, United States, 2011–2016. Am J Obstet Gynecol. 2021;225(2):183.e1‐183.e16. doi:10.1016/j.ajog.2021.02.028 PMC11697984 33640361

[6] Kozhimannil KB . Indigenous maternal health—a crisis demanding attention. JAMA Health Forum. 2020;1(5):e200517. doi:10.1001/jamahealthforum.2020.0517 36218496

[7] Trost S , Busacker A , Leonard M . Pregnancy‐related deaths: data from Maternal Mortality Review Committees in 38 U.S. States, 2020 . CDC: Maternal Mortality Prevention; May 24, 2024. Accessed May 28, 2024. https://www.cdc.gov/maternal‐mortality/php/data‐research/2020‐mmrc.html

[8] Trost S , Beauregard J , Chandra G , et al. Pregnancy‐related deaths among American Indian or Alaska Native persons: data from Maternal Mortality Review Committees in 36 US States, 2017–2019 . CDC: Maternal Mortality Prevention. September 19, 2023. Accessed December 1, 2023. https://www.cdc.gov/maternal‐mortality/php/data‐research/2017‐2019‐aian.html

[9] Trost S , Beauregard J , Chandra G , et al. Pregnancy‐related deaths: data from Maternal Mortality Review Committees in 36 US States, 2017–2019 . CDC: Maternal Mortality Prevention. September 26, 2022. Accessed December 1, 2023. https://www.cdc.gov/maternal‐mortality/php/data‐research/mmrc‐2017‐2019.html

[10] CDC, Pregnancy Mortality Surveillance System . Updated Pregnancy Mortality Surveillance System data to include 2020 . CDC: Maternal Mortality Prevention. May 20, 2024. Accessed June 24, 2024. https://www.cdc.gov/maternal‐mortality/php/pregnancy‐mortality‐surveillance/index.html

[11] Hung P , Henning‐Smith CE , Casey MM , Kozhimannil KB . Access to obstetric services in rural counties still declining, with 9 percent losing services, 2004–14. Health Aff. 2017;36(9):1663‐1671. doi:10.1377/hlthaff.2017.0338 28874496

[12] Hung P , Kozhimannil KB , Casey MM , Moscovice IS . Why are obstetric units in rural hospitals closing their doors? Health Serv Res. 2016;51(4):1546‐1560. doi:10.1111/1475-6773.12441 26806952 PMC4946037

[13] Handley SC , Passarella M , Herrick HM , et al. Birth volume and geographic distribution of US hospitals with obstetric services from 2010 to 2018. JAMA Netw Open. 2021;4(10):e2125373. doi:10.1001/jamanetworkopen.2021.25373 34623408 PMC8501399

[14] Handley SC , Passarella M , Interrante JD , Kozhimannil KB , Lorch SA . Perinatal outcomes for rural obstetric patients and neonates in rural‐located and metropolitan‐located hospitals. J Perinatol. 2022;42(12):1600‐1606. doi:10.1038/s41372-022-01490-7 35963889 PMC12832148

[15] Brigance C , Lucas R , Jones E , et al. Nowhere to Go: Maternity Care Deserts Across the U.S. (Report No. 3) . March of Dimes; 2022. Accessed October 21, 2022. https://www.marchofdimes.org/research/maternity‐care‐deserts‐report.aspx

[16] Anonymous . Rural Hospitals at Risk of Closing Report . Center for Healthcare Quality & Payment Reform; 2024. Accessed April 29, 2024. https://ruralhospitals.chqpr.org/downloads/Rural_Hospitals_at_Risk_of_Closing.pdf

[17] Topchik M , Balfour B , Brown T , Wiesse A , Pinette M . Rural America's OB Deserts Widen in Fallout from Pandemic . Chartis; 2023. Accessed April 29, 2024. https://www.chartis.com/sites/default/files/documents/rural_americas_ob_deserts_widen_in_fallout_from_pandemic_12‐19‐23.pdf

[18] Kozhimannil KB , Hung P , Henning‐Smith C , Casey MM , Prasad S . Association between loss of hospital‐based obstetric services and birth outcomes in rural counties in the United States. JAMA. 2018;319(12):1239. doi:10.1001/jama.2018.1830 29522161 PMC5885848

[19] Thorsen ML , Harris S , McGarvey R , Palacios J , Thorsen A . Evaluating disparities in access to obstetric services for American Indian women across Montana. J Rural Health. 2022;38(1):151‐160. doi:10.1111/jrh.12572 33754411 PMC8458487

[20] Thorsen ML , Harris S , Palacios JF , McGarvey RG , Thorsen A . American Indians travel great distances for obstetrical care: examining rural and racial disparities. Soc Sci Med. 2023;325:115897. doi:10.1016/j.socscimed.2023.115897 37084704 PMC10164064

[21] Centers for Medicare & Medicaid Services (CMS) . Emergency Medical Treatment & Labor Act (EMTALA) . Accessed April 17, 2022. https://www.cms.gov/Regulations‐and‐Guidance/Legislation/EMTALA

[22] Macones GA , Pettker C , Mascola MA , Heine RP , Committee on Obstetric Practice. Hospital‐Based Triage of Obstetric Patients. American College of Obstetricians and Gynecologists; 2016. Accessed July 12, 2024. https://www.acog.org/clinical/clinical‐guidance/committee‐opinion/articles/2016/07/hospital‐based‐triage‐of‐obstetric‐patients

[23] Kozhimannil KB , Interrante JD , Tuttle MS , Gilbertson M , Wharton KD . Local capacity for emergency births in rural hospitals without obstetrics services. J Rural Health. 2021;37(2):385‐393. doi:10.1111/jrh.12539 33200829

[24] World Health Organization . Monitoring Emergency Obstetric Care: A Handbook . WHO; 2009. Accessed April 12, 2022. https://www.who.int/publications/i/item/9789241547734

[25] Ebener S , Stenberg K , Brun M , et al. Proposing standardised geographical indicators of physical access to emergency obstetric and newborn care in low‐income and middle‐income countries. BMJ Glob Health. 2019;4(Suppl5):e000778. doi:10.1136/bmjgh-2018-000778 PMC6623986 31354979

[26] Holman C , Glover AL , Fertaly K , Nelson M . Montana Levels of Care Assessment Tool (LOCATe) Statewide Report . University of Montana Rural Institute for Inclusive Communities; 2022. Accessed March 3, 2022. https://www.mtmoms.org/levels‐of‐care‐assessment‐tool‐locate/

[27] Holman C , Glover A , Fertaly K , Nelson M . Operationalizing risk‐appropriate perinatal care in a rural US State: directions for policy and practice. BMC Health Serv Res. 2023;23(1):601. doi:10.1186/s12913-023-09552-y 37291539 PMC10251618

[28] Catalano A , Bennett A , Busacker A , et al. Implementing CDC's Level of Care Assessment Tool (LOCATe): a national collaboration to improve maternal and child health. J Womens Health. 2017;26(12):1265‐1269. doi:10.1089/jwh.2017.6771 PMC6020827 29240547

[29] Kilpatrick SJ , Menard MK , Zahn CM , Callaghan WM . Obstetric Care Consensus #9: levels of maternal care. Am J Obstet Gynecol. 2019;221(6):B19‐B30. doi:10.1016/j.ajog.2019.05.046 31351999

[30] Centers for Disease Control & Prevention, Division of Reproductive Health . CDC Levels of Care Assessment Tool (CDC LOCATe) . CDC: Maternal Infant Health. June 16, 2021. Accessed April 13, 2022. https://www.cdc.gov/reproductivehealth/maternalinfanthealth/cdc‐locate/index.html

[31] Anonymous . ACOG Obstetric Care Consensus No. 9: levels of maternal care: correction. Obstet Gynecol. 2023;141(4):864. doi:10.1097/AOG.0000000000005128 36961972

[32] Committee on Fetus & Newborn , Barfield WD , Papile LA , et al. Levels of neonatal care. Pediatrics. 2012;130(3):587‐597. doi:10.1542/peds.2012-1999 22926177

[33] Harris PA , Taylor R , Thielke R , Payne J , Gonzalez N , Conde JG . Research electronic data capture (REDCap)—a metadata‐driven methodology and workflow process for providing translational research informatics support. J Biomed Inform. 2009;42(2):377‐381. doi:10.1016/j.jbi.2008.08.010 18929686 PMC2700030

[34] Harris PA , Taylor R , Minor BL , et al. The REDCap Consortium: building an international community of software platform partners. J Biomed Inform. 2019;95:103208. doi:10.1016/j.jbi.2019.103208 31078660 PMC7254481

[35] Google . Google Maps distance calculations between hospitals without obstetrics units to various levels of maternal and neonatal care . Accessed December 11, 2023. https://www.google.com/maps

[36] US Census Bureau . Office of Management and Budget: Metropolitan and Micropolitan Statistical Areas . US Census Bureau; 2020. Accessed July 11, 2024. https://www.census.gov/programs‐surveys/metro‐micro.html

[37] Ingram DD , Franco SJ . 2013 NCHS urban‐rural classification scheme for counties. Vital Health Stat 2. 2014(166):1‐73. 24776070

[38] US Department of Agriculture, Economic Research Service . Rural‐Urban Continuum Codes . Accessed July 11, 2024. https://www.ers.usda.gov/data‐products/rural‐urban‐continuum‐codes.aspx

[39] StataCorp . Stata Statistical Software: Release 18. StataCorp, LLC; 2023.

[40] Gausman J , Pingray V , Adanu R , et al. Validating indicators for monitoring availability and geographic distribution of emergency obstetric and newborn care (EmoNC) facilities: a study triangulating health system, facility, and geospatial data. PLoS ONE. 2023;18(9):e0287904. doi:10.1371/journal.pone.0287904 37708180 PMC10501555

[41] Sikder SS , Labrique AB , Ullah B , et al. Accounts of severe acute obstetric complications in Rural Bangladesh. BMC Pregnancy Childbirth. 2011;11(1):76. doi:10.1186/1471-2393-11-76 22018330 PMC3250923

[42] Espinoza J , Vidaeff A , Pettker C , Simhan H . Gestational hypertension and preeclampsia: ACOG Practice Bulletin, Number 222. Obstet Gynecol. 2020;135(6):e237. doi:10.1097/AOG.0000000000003891 32443079

[43] Tita Alan TN , Carlo Waldemar A , McClure Elizabeth M , et al. Azithromycin to prevent sepsis or death in women planning a vaginal birth. N Engl J Med. 2023;388(13):1161‐1170. doi:10.1056/NEJMoa2212111 36757318 PMC10627427

[44] Boddu PK , Velumula PK , Jani S , et al. Neonatal resuscitation program (NRP) guidelines and timing of major resuscitation events in delivery rooms at a level III NICU: understanding deviations. Resusc Plus. 2024;17:100571. doi:10.1016/j.resplu.2024.100571 38419829 PMC10900917

[45] World Health Organization . WHO Recommendation on Routes of Oxytocin Administration for the Prevention of Postpartum Haemorrhage after Vaginal Birth. World Health Organization; 2020. Accessed May 16, 2024. https://www.ncbi.nlm.nih.gov/books/NBK564752/ 33252891

[46] Antonella G , Luisa DB , Chiara A , Alessandra R , Caserta D . Conservative and timely treatment in retained products of conception: a case report of placenta accreta ritention. Int J Clin Exp Pathol. 2015;8(10):13625‐13629. 26722586 PMC4680531

[47] Sellmyer MA , Desser TS , Maturen KE , Jeffrey RB , Kamaya A . Physiologic, histologic, and imaging features of retained products of conception. RadioGraphics. 2013;33(3):781‐796. doi:10.1148/rg.333125177 23674774

[48] American College of Obstetricians & Gynecologists . Blood Bank: Massive Transfusion Protocol (MTP) . Accessed February 22, 2024. https://www.acog.org/‐/media/project/acog/acogorg/files/forms/districts/smi‐ob‐hemorrhage‐bundle‐poster‐massive‐transfusion‐protocol.pdf

[49] McWilliams GDE , Hill MJ , Dietrich CS . Gynecologic emergencies. Surg Clin N Am. 2008;88(2):265‐283. doi:10.1016/j.suc.2007.12.007 18381113

[50] Montana EMS, Trauma Systems & Injury Prevention Program, Montana Department of Public Health & Human Services . Montana Injury Prevention Annual Report . Accessed June 19, 2024. https://dphhs.mt.gov/assets/publichealth/EMSTS/Data/IPAnnualReport2020Data.pdf

[51] Ameh CA , Mdegela M , White S , van den Broek N . The effectiveness of training in emergency obstetric care: a systematic literature review. Health Policy Plan. 2019;34(4):257‐270. doi:10.1093/heapol/czz028 31056670 PMC6661541

[52] Forman J , Henkle J . Obstetrical readiness: preparing rural emergency departments without hospital‐based obstetrical services. Clin Obstet Gynecol. 2022;65(4):829. doi:10.1097/GRF.0000000000000749 36162083

[53] Thenuwara K , Dunbar A . Using simulation to improve and maintain obstetrical skills in rural hospitals. Clin Obstet Gynecol. 2022;65(4):817. doi:10.1097/GRF.0000000000000741 36044624

[54] Thenuwara K , Santillan D , Henkle J , et al. A statewide mobile simulation program for improving obstetric skills in rural hospitals. Anesth Analg. 2024;139(5):931‐939. doi:10.1213/ANE.0000000000006883 38758671

[55] Alliance for Innovation on Maternal Health . AIM Obstetric Emergency Readiness Resource Kit . Accessed July 12, 2024. https://saferbirth.org/aim‐obstetric‐emergency‐readiness‐resource‐kit/

[56] Fertaly K , Javorka M , Brown D , Holman C , Nelson M , Glover A . Obstetric transport in rural settings: referral and transport of pregnant patients in a state without a perinatal regionalized system of care. Health Serv Res. 2024;59(5):e14365. doi:10.1111/1475-6773.14365 39103196 PMC11366954

[57] Alliance for Innovation on Maternal Health . Strategies for Implementation of Regionalized Risk‐Appropriate Maternal Care on a National Scale . AIM; 2022. Accessed July 12, 2024. https://saferbirth.org/wp‐content/uploads/10‐18‐2022‐FINAL_AIM_HRSA‐LoMC‐Report.pdf

[58] American College of Obstetricians & Gynecologists . Committee Opinion No. 590: preparing for clinical emergencies in obstetrics and gynecology. Obstet Gynecol. 2014;123(3):722. doi:10.1097/01.AOG.0000444442.04111.c6 24553170

[59] National Advisory Committee on Rural Health and Human Services . Maternal and Obstetric Care Challenges in Rural America: Policy Brief and Recommendations to the Secretary. Health Resources and Services Administration; 2020. Accessed July 12, 2024. https://www.hrsa.gov/sites/default/files/hrsa/advisory‐committees/rural/publications/2020‐maternal‐obstetric‐care‐challenges.pdf

[60] Kunz SN , Phibbs CS , Profit J . The changing landscape of perinatal regionalization. Sem Perinatol. 2020;44(4):151241. doi:10.1016/j.semperi.2020.151241 32248957

[61] Van Otterloo LR , Connelly CD . Risk‐appropriate care to improve practice and birth outcomes. J Obstet Gynecol Neonatal Nurs. 2018;47(5):661‐672. doi:10.1016/j.jogn.2018.05.004 30196808

[62] Ryan GM . Toward improving the outcome of pregnancy: Recommendations for the regional development of perinatal health services. Obstet Gynecol. 1975;46(4):375‐384. 1165870

[63] Johnson KG . The promise of regional perinatal care as a national strategy for improved maternal and infant care. Public Health Rep. 1982;97(2):134‐139. 7063594 PMC1424302

