# Supplementary material for: Obstetric care in rural critical access hospitals: A domestic application of the World Health Organization Emergency Obstetric Care framework in rural communities
Source: J Rural Health. 2025 May 24;41(2):e70037. doi: 10.1111/jrh.70037 (PMC12102685; doi:10.1111/jrh.70037)
Supplement: Supplementary file 1 — Supporting Information [file JRH-41-0-s001.docx]

# Supporting Information

# Appendix

## World Health Organization (WHO) Emergency Obstetric Care (EmOC) Indicator Tables

**Table S1** Wording Adjustments of WHO EmOC Indicators

| **Original Wording of WHO EmOC Indicators** | **Adapted Wording of WHO EmOC Indicators for Survey** |
| --- | --- |
| Administer parenteral antibiotics | Administer intravenous or injection antibiotics to both mothers and infants |
| Administer uterotonic drugs | (Same) Administer uterotonic drugs |
| Administer parenteral anticonvulsants for preeclampsia and eclampsia | Administer magnesium sulfate for severe preeclampsia and eclampsia |
| Manually remove the placenta | Manually remove a placenta |
| Remove retained products | Remove retained products of delivery |
| Perform assisted vaginal delivery | Perform assisted vaginal delivery with a soft cup vacuum extractor |
| Perform basic neonatal resuscitation | (Same) Perform basic neonatal resuscitation |
| Perform surgery | Perform surgery (e.g., cesarean section) |
| Perform blood transfusion | Conduct blood transfusion |
| Not included | Provide and interpret fetal heart tracing in an emergency setting |
| Not included | Plan or policy for an emergency cesarean in the case of a life-threatening obstetrical hemorrhage or profound fetal distress |
| Not included | Ultrasound availability for obstetrics |

**Table S2** WHO EmOC Indicator: Conduct Blood Transfusion

| **My hospital has the capacity to… Conduct blood transfusion (N=34)** | **n (%)** |
| --- | --- |
| Yes | 26 (76.5) |
| No - we lack the necessary staff, and current staff lack appropriate training and experience | 0 (0.0) |
| No - we lack the necessary equipment (including lab services and blood products) | 3 (8.8) |
| No- we lack both staff and equipment | 4 (11.8) |
| Don’t know | 1 (2.9) |

**Table S3** WHO EmOC Indicator: Administer Magnesium Sulfate for Severe (Pre-)Eclampsia

| **My hospital has the capacity to… Administer magnesium sulfate for severe preeclampsia and eclampsia (N=34)** | **n (%)** |
| --- | --- |
| Yes | 29 (85.3) |
| No - we lack the necessary staff, and current staff lack appropriate training and experience | 3 (8.8) |
| No - we lack the necessary equipment (including lab services and blood products) | 0 (0.0) |
| No- we lack both staff and equipment | 0 (0.0) |
| Don’t know | 2 (5.9) |

**TablE S4** WHO EmOC Indicator: Administer Uterotonic Drugs

| **My hospital has the capacity to… Administer uterotonic drugs (N=34)** | **n (%)** |
| --- | --- |
| Yes | 17 (50.0) |
| No - we lack the necessary staff, and current staff lack appropriate training and experience | 5 (14.7) |
| No - we lack the necessary equipment (including lab services and blood products) | 1 (2.9) |
| No- we lack both staff and equipment | 7 (20.6) |
| Don’t know | 4 (11.8) |

**Table S5** WHO EmOC Indicator: Perform Assisted Vaginal Delivery

| **My hospital has the capacity to… Perform assisted vaginal delivery with a soft cup vacuum extractor (N=34)** | **n (%)** |
| --- | --- |
| Yes | 5 (14.7) |
| No - we lack the necessary staff, and current staff lack appropriate training and experience | 5 (14.7) |
| No - we lack the necessary equipment (including lab services and blood products) | 4 (11.8) |
| No- we lack both staff and equipment | 19 (55.9) |
| Don’t know | 1 (2.9) |

**Table S6** WHO EmOC Indicator: Manually Remove Placenta

| **My hospital has the capacity to… Manually remove a placenta (N=34)** | **n (%)** |
| --- | --- |
| Yes | 5 (14.7) |
| No - we lack the necessary staff, and current staff lack appropriate training and experience | 11 (32.4) |
| No - we lack the necessary equipment (including lab services and blood products) | 2 (5.9) |
| No- we lack both staff and equipment | 14 (41.9) |
| Don’t know | 2 (5.9) |

**Table S7** WHO EmOC Indicator: Remove Retained Products

| **My hospital has the capacity to… Remove retained products (N=30)** | **n (%)** |
| --- | --- |
| Yes | 4 (11.8) |
| No - we lack the necessary staff, and current staff lack appropriate training and experience | 9 (26.5) |
| No - we lack the necessary equipment (including lab services and blood products) | 2 (5.9) |
| No- we lack both staff and equipment | 17 (50.0) |
| Don’t know | 2 (5.9) |

**Table S8** WHO EmOC Indicator: Administer antibiotics

| **My hospital has the capacity to… Administer intravenous or injection antibiotics to both mothers and infants (N=34)** | **n (%)** |
| --- | --- |
| Yes | 29 (85.3) |
| No - we lack the necessary staff, and current staff lack appropriate training and experience | 1 (2.9) |
| No - we lack the necessary equipment (including lab services and blood products) | 0 (0.0) |
| No- we lack both staff and equipment | 2 (5.9) |
| Don’t know | 2 (5.9) |

**Table S9** WHO EmOC Indicator: Perform Surgery

| **My hospital has the capacity to…**  **Perform surgery (e.g., cesarean section) (N=34)** | **n (%)** |
| --- | --- |
| Yes | 0 (0.0) |
| No - we lack the necessary staff, and current staff lack appropriate training and experience | 9 (26.5) |
| No - we lack the necessary equipment (including lab services and blood products) | 0 (0.0) |
| No- we lack both staff and equipment | 24 (70.6) |
| Don’t know | 1 (2.9) |

**Table S10** WHO EmOC Indicator: Neonatal Resuscitation

| **My hospital has the capacity to… Perform basic neonatal resuscitation (N=34)** | **n (%)** |
| --- | --- |
| Yes | 28 (82.4) |
| No - we lack the necessary staff, and current staff lack appropriate training and experience | 4 (11.8) |
| No - we lack the necessary equipment (including lab services and blood products) | 0 (0.0) |
| No- we lack both staff and equipment | 2 (5.9) |
| Don’t know | 0 (0.0) |
